# Critical soft skill competencies that clinical nurse educators consider important to evaluate in nurses

**DOI:** 10.1002/nop2.70047

**Published:** 2024-10-01

**Authors:** Youngkwan Song, Cynthia M. Lafond, Catherine Vincent, Mi Ja Kim, Chang G. Park, Linda L. McCreary

**Affiliations:** ^1^ Division of Endocrinology, Diabetes, and Metabolism, Department of Medicine University of Illinois at Chicago Chicago Illinois USA; ^2^ Nursing Center of Excellence, Ascension St. Louis Missouri USA; ^3^ College of Nursing University of Illinois at Chicago Chicago Illinois USA

**Keywords:** assessment, clinical nursing education, competency, soft skill, qualitative study

## Abstract

**Aim:**

Guided by Benner's framework, From Novice to Expert, this study aimed to identify (1) critical soft skills to be evaluated in nurses and (2) levels of nursing behaviour indicating achievement of soft skills to provide a framework for developing a soft skills rubric.

**Background/Introduction:**

Nurse shortages are often attributable to high turnover rates among nurses. To improve this situation, healthcare facilities implement transition programmes and continuing education with a primary focus on developing and maintaining nurses' knowledge and performance (hard skills). However, balancing hard and soft skills (beliefs, values and attitudes) is key to nurses' quality of care and ultimately to their retention. Despite the importance of soft skills, their intangible nature creates problems in evaluating nurses' attainment of these skills and in providing constructive feedback to help them set concrete goals for improving their practice.

**Methods:**

This qualitative descriptive study described critical soft skills in the nursing context. A purposeful sampling strategy was used to enrol 13 clinical nurse educators from multiple hospital units; each had more than 2 years of clinical nurse educator experience and had completed preceptor training. One‐to‐one interviews with these individuals were audio recorded, transcribed and subjected to direct content analysis using NVivo 12. The study follows the Consolidated criteria for reporting qualitative research (COREQ) guidelines for qualitative studies.

**Findings:**

Five main competencies, comprising 20 subcompetencies, were identified: personal growth, effective interactions, professionalism, teamwork and the caring role. For each subcompetency, four skill levels were delineated using clinical indicators.

**Conclusion:**

This study's findings can enhance understanding of clinical nurse educators' perceptions regarding soft skills required of nurses.

**Implications for the profession and/or patient care:**

The soft skills identified can be incorporated into a rubric to be used by clinical nurse educators to evaluate and guide nurses' professional development and contribute to improving quality of care.

No patient or public contribution is required for this study.

## 1 INTRODUCTION

Nurses are crucial human resources within the healthcare system of every country. They deliver front‐line care and contribute to optimal patient outcomes by providing quality care. Despite the need for nurses in healthcare systems, the current number of active nurses falls significantly short of the demand (Drennan & Ross, 2019). The ageing of the nursing workforce with the consequent retirements and the high number of nurses otherwise leaving the profession place many countries at risk of serious nursing shortages. The American healthcare system in particular is suffering from a serious, ongoing nurse shortage that is primarily attributable to a high turnover rate among nurses (15.9%), as well as new graduate nurses (NGN), in their first year on the job (21.2%) (Nursing Solutions Inc., 2020). This shortage of nurses negatively impacts both delivery and quality of care, leading to adverse patient outcomes (Cho et al., 2015). Previous studies revealed that high turnover intention and actual turnover among nurses are often due to their perceived low level of nursing competency, low‐performance level and lack of preparedness for practice (Cheng et al., 2014; Kaihlanen et al., 2020). To improve nurse retention and address the nurse shortage, many healthcare facilities implement transition programmes with preceptorship and continuing education that focus on developing and maintaining nurses' required competencies for direct care. These programmes aim to support NGNs' successful transition to practice and nurse retention (L'Ecuyer et al., 2018). However, the problems surrounding retention and the nurse shortage seem to persist, and this situation is attributable to the singular focus on competencies for direct care, which could neglect other important aspects of nursing practice (Hires, 2018).

The nursing discipline recognises that nurses' crucial role of delivering quality care on the front lines is fulfilled not only by their clinical performance but also by their commitment to quality patient care (Miedaner et al., 2018). Herein, ‘performance’ refers to execution of the ‘hard skills’—that is, what nurses know and do; traditional nursing education has prioritised performance to date. ‘Commitment to quality patient care’ refers to the ‘soft skills’ that constitute who nurses are because they are rooted in beliefs, values and attitudes. Soft skills encompass, for example, self‐awareness, flexibility, communication, critical thinking and even diplomacy skills, that influence employees' adaptation to the workplace (Haselberger et al., 2012).

In the nursing context, self‐assessment revealed that nurses' main concerns involved soft skill competency areas such as communication, critical thinking, teamwork, helping roles and professionalism (Song & McCreary, 2020). Such skills contribute to employee productivity, job satisfaction and healthy workplace culture (Lepeley et al., 2021). Given the holistic nature of nursing, achieving a balance of hard and soft skill competencies is significant not only for nurses to attain excellence in patient care and for improving job retention but also extends to staff development, service monitoring and beyond.

Despite the importance of soft skill competencies in nursing, their intangible nature has created gaps, such as the lack of objective assessment tools, making it problematic to accurately evaluate the acquisition of these skills (Lombarts et al., 2014) and to provide constructive feedback to help nurses establish concrete goals for improving their practice. Although soft skill competencies are not directly observable, they can often be inferred from nurses' words and actions (e.g., their behaviours) (Bednar & Levie, 1993). Given these considerations, the development of an objective tool—such as a rubric—can fill the assessment gap by measuring nurses' soft skill competencies based on specific clinical indicators (Song & McCreary, 2020). Therefore, this study aimed to identify (1) critical soft skills to be evaluated in nurses and (2) levels of nursing behaviour indicating achievement of soft skills in order to provide a framework for developing a soft skills rubric.

## 2 METHOD

### 2.1 Design

This study employed a qualitative descriptive approach to comprehensively describe critical soft skill competencies that clinical nurse educators (CNE) consider important to evaluate in nurses. The study was conducted following the COREQ guidelines for qualitative studies.

### 2.2 Participants and setting

Purposeful sampling was used to enrol 13 interviewees who met these inclusion criteria: CNEs who (1) spoke English, (2) held a bachelor's degree in nursing or higher, (3) had more than 2 years of experience as a CNE during NGN orientation and (4) had completed preceptor training. Enrolment continued until no new information could be obtained and further coding was not feasible, indicating that data saturation had been achieved (Fusch & Ness, 2015). Sampling and data collection were performed in a university‐affiliated hospital. The first author visited unit staff meetings to present the study, invited study participation and distributed an IRB‐approved study flyer containing the first author's contact information. The flyer was (1) posted in unit break rooms and other shared spaces likely to be visited by potential participants and (2) sent to CNEs via email.

### 2.3 Theoretical framework

Benner's (2001) framework, From Novice to Expert, introduces a progression in the development of a nurse over a given timeframe and outlines five levels of nursing competence: novice, advanced beginner, competent, proficient and expert. Guided by this developmental framework, we sought to identify critical soft skills in nursing and develop descriptors of behaviours indicative of varying competency levels. However, adaptations regarding level labelling were inevitable to more realistically reflect the clinical nursing practice context. For example, Benner's five levels of nursing competence begin with the ‘novice’ level, which refers to student nurses. Given that this study focuses on evaluations performed by CNEs in practice, we excluded this level from our study. Also, we found Benner's ‘advanced beginner’ level to be misleading for evaluators due to the nuances of ‘advanced’, so we relabelled it as ‘beginning’ for the lowest level. Similarly, we modified ‘competent’ to ‘developing’, ‘proficient’ to ‘accomplished’ and ‘expert’ to ‘exemplary’.

Given that most competency assessments are conducted during the first years of practice, the behaviour indicators to be produced for all four levels in this study would be most useful for helping NGNs understand the behaviours expected of them both at the present time and throughout their career. This understanding could in turn help NGNs to establish self‐improvement goals, thus easing their transition to the realities of practice. In addition, nursing competency assessments can be conducted at several key stages throughout a nurse's career to ensure that they meet the required standards for providing safe and effective care. Therefore, we have also factored this consideration into our rubric to make it applicable to all levels of nurses.

Furthermore, soft skill competencies introduced in a previously published literature review (Song & McCreary, 2020) provided a basis for the study interview guide and its probing questions as well as for the data analysis.

### 2.4 Data collection

One‐to‐one interviews were conducted in person with 13 RNs with a broad range of CNE experience. All interviews were performed by in a private setting by the first author, who had no supervisory authority over the participants. The 60‐ to 90‐min interviews followed a semi‐structured guideline that included the following questions: ‘What critical competencies do you regard as important to be evaluated in nurses?’, ‘In your own words, among the [supplied] list of competencies, what does this competency mean or involve?’ and ‘What evidence would indicate that a nurse has achieved this competency?’. Probing questions that allowed for follow‐up on unanticipated responses were also used. Additionally, the first author utilised a blank rubric template with four rows to indicate the four levels of skills and a column for behaviour indicators for each competency, which were added as a field note. This approach facilitated participants' ability to gradate differences across the four levels. One‐to‐one interviews were chosen over focus groups to protect participant confidentiality and to allow more in‐depth information to be gathered from each individual.

### 2.5 Data analysis

Interview data were transcribed and analysed following the directed content analysis method (Hsieh & Shannon, 2005) using NVivo version 12 software. Selected qualitative data were identified through multiple readings of transcripts; initial competencies were identified and expanded as new competencies emerged. Passages related to each competencies were extracted, clustered and labelled with a code. Codes were grouped into competencies and subcompetencies. For each subcompetency, four skill levels were then delineated and illustrated with relevant quotations. Across all subcompetencies, the analysis included comparison of soft skill competencies derived from an earlier literature review (Song & McCreary, 2020).

### 2.6 Ethical considerations

Institutional review board approval of the study was granted at the university‐affiliated hospital (REDACTED) as well as the authors' home institution (REDACTED). Signed informed consent was obtained from all interviewees before they completed a brief demographic questionnaire and an audio‐recorded interview in a private room. The interviewees were assured that their study participation would be known only to the first author and would have no bearing on their relationship with the university‐affiliated hospital; they were also informed that if they were to be quoted in the study documentation, pseudonyms would be substituted for their names to protect their confidentiality. All information provided by participants was kept confidential, and each participant's information was labelled with a unique ID number instead of their name.

### 2.7 Rigour and trustworthiness

Trustworthiness of study findings was enhanced by following stringent criteria suggested by Lincoln and Guba (1985). To strengthen *credibility*, prolonged engagement with the setting was fulfilled by the first author's (investigator's) 12‐week research internship as well as member checking and peer debriefing. Immediate member checking of interviewee responses was performed during data collection to allow participants to confirm or correct the first author's interpretations of their statements. In addition, peer debriefing included checking of each transcript's content by an expert co‐author and discussion of any differing opinions between the first and co‐authors until a consensus was reached. To enhance *dependability*, rich description of study methods was carried out by preparing detailed drafts according to the study protocol. Moreover, the investigator established an audit trail by developing a detailed record of the data collection process. Coding accuracy was confirmed by having an external coder independently code two of the 13 transcripts. High inter‐coder reliability (Transcript 1 score: 88.5 and Transcript 2 score: 98.7; average: 93.4) supported the overall dependability of the study. To support *confirmability*, all data collection and analysis decisions were made in peer debriefings that included the first author and a co‐author experienced in qualitative research.

Use of an adapted version of the triangulation protocol method (Adams et al., 2015; Farmer et al., 2006) also strengthened trustworthiness. In this study, an earlier literature review (Song & McCreary, 2020) provided a basis for the study interview guide and its probing questions. In addition, data analysis was conducted through constant comparison of findings between the literature review and the analysis of qualitative data collected from interviewees working across multiple units. Interpretation and integration of key findings from 16 empirical studies and 13 interviews resulted in identification of essential competencies. Finally, study *transferability* was augmented by purposeful sampling of experts in the phenomena of interest and interviewing participants from multiple units.

## 3 FINDINGS

**FIGURE 1:**
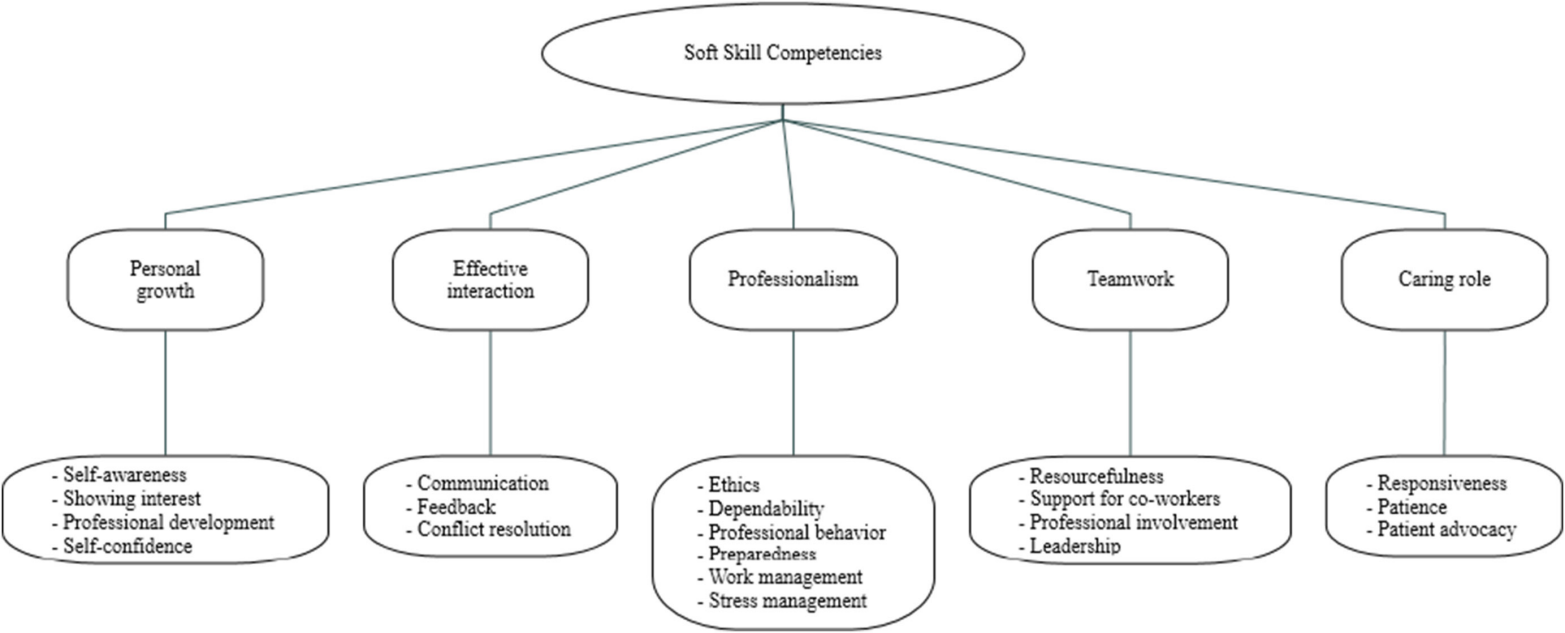
Main competencies and subcompetencies of critical soft skill competencies.

Twelve females and one male (*N* = 13) participated in this study. Their ages ranged from 26 to 61 years, and their experience as a CNE ranged from 2 to 14 years. The participants represented eight different units; four participants had a master's degree, nine had a Bachelor of Science in Nursing (BSN) degree and six of the nine BSN holders were working on a Doctor of Nursing Practice or Master of Science in Nursing degree. Analysis of data generated the five major competencies and 20 subcompetencies outlined in Figure 1. Interviewees described various levels of nurses' soft skill competencies. From their descriptions, four levels of performance were distilled for each subcompetency. For each main competency presented below, subcompetencies are briefly defined and illustrated with the most relevant and representative interview quotations. Table 1 shows the definitions and descriptors identified based on the qualitative data.

**TABLE 1:** Definitions and descriptors of identified critical soft skill competencies in nurses.

Competency/Subcompetency	Definitions/Descriptors
1. Personal growth: showing commitment to learning and professional growth, awareness of their own limitations and self‐confidence in their abilities	
Self‐awareness: Nurse recognises own abilities and limitations and can express personal needs and boundaries	
Beginning	Nurse does not realistically perceive own abilities and limitations; may not speak up if assigned work is beyond personal skill level; struggles to prove herself/himself in care that surpass capabilities
Developing	Nurse is becoming able to articulate difficulties and seeks opportunities to overcome weaknesses; tries to complete the assigned work while expressing concerns about own ability; is starting to know limits of safe patient care she/he can safely perform
Accomplished	Nurse knows own strengths and weaknesses and points out an inappropriate assignment; usually knows how much time care takes and can decide how much work to accept; identifies resources to overcome limitations
Exemplary	Nurse comprehensively understands own role and ability; is aware of the kinds of patients and care that she/he can manage well and consults leadership to request necessary work adjustments based on own needs and values
Showing interest: Nurse is willing to ask questions when necessary and expresses interest in learning about things in the workplace.	
Beginning	Nurse may hesitate to ask questions about things not understood; may not show interest in learning how to improve skills or patient care
Developing	Nurse does not hesitate to admit what is not known or to ask questions to prevent errors; shows interest in learning how to improve own work
Accomplished	Nurse asks questions and seeks feedback to improve own practice; seeks out opportunities to learn
Exemplary	Nurse consistently asks questions about how own practice in the unit can be enhanced; communicates curiosity and excitement about expanding own practice
Professional development: nurse is motivated to expand own roles and seeks learning opportunities to develop knowledge and skills	
Beginning	Nurse takes part in the minimum required trainings/continuing education to maintain licensure; may require prompting to take part in professional development opportunities
Developing	Nurse is beginning to seek out continuing education in specialty to independently develop own practice; may attend conferences or workshops to gain advanced levels of nursing skills and knowledge
Accomplished	Nurse may earn a certificate or advanced degree in a specialty area; uses professional resources and shares what is learned to improve quality of care in workplace
Exemplary	Nurse takes the initiative in professional development by earning one or more certificates or advanced degrees in own specialty area; may present at conferences or take on roles such as clinical educator or preceptor
Self‐confidence: Nurse demonstrates assurance based on accurate assessment of their ability to independently complete job tasks	
Beginning	Nurse is hesitant to ask questions or try something new on her/his own and needs frequent and extensive direction; relies on others to give direction
Developing	Nurse can carry out most care without prompting but may need to be reassured at times; attempts to solve problems but may need guidance; starting to establish a professional, competent identity.
Accomplished	Nurse has developed the necessary job skills and functions confidently without supervision; speaks with conviction and identifies and shares solutions to problems
Exemplary	Nurse is self‐directed, comfortable and assertive in their role and takes responsibility for own actions; is proactive and takes appropriate actions without hesitation; actions and tone of voice reflect self‐assurance
2. Effective interaction: Exchanging information clearly and accurately, acting on constructive feedback and resolving conflicts	
Communication: Nurse clearly and accurately exchanges information and interacts effectively with others.	
Beginning	Nurse may be unable to clearly and completely articulate own observations verbally or in writing; may neglect to communicate important information
Developing	Nurse is able, verbally and in writing, to accurately describe a patient's condition to all members of the care team
Accomplished	Nurse is confident in communicating verbally and in writing with others; includes all relevant information and offers possible causes or suggestions when reporting on a patient's condition
Exemplary	Nurse communicates professionally with others in understandable language; focuses during conversations and uses nonverbal communication effectively; nurse's interactions model how to create a positive culture in order to meet mutual goals
Feedback: Nurse actively seeks input from patients and staff regarding own performance and uses that feedback to build on strengths and improve performance	
Beginning	Nurse receives feedback but may not change behaviour; may react defensively or become frustrated
Developing	Nurse responds to constructive feedback and tries to improve even if she/he does not agree with it; responds less emotionally to constructive feedback
Accomplished	Nurse accepts and recognises the usefulness of feedback; asks for feedback and is willing to discuss it to improve own practice
Exemplary	Nurse seeks and applies feedback to further develop own practice and achieve better patient outcomes; accepts constructive criticism without becoming defensive
Conflict resolution: Nurse manages difficult situations and resolves conflicts diplomatically	
Beginning	Nurse is sometimes unable to demonstrate an ability to address conflict; may lack strategies to manage own emotions during difficult encounters
Developing	Nurse may respond to conflicts indirectly by complaining, which may not resolve conflicts effectively; may have developed strategies to manage negative emotions; and move past the difficulty quickly
Accomplished	Nurse responds directly to conflict by respectfully discussing it and by collaboratively planning action; resolves issues while maintaining working relationships and refrains from expressing negative emotions
Exemplary	Nurse manages conflict and resolves the entire problem directly and effectively; explores the other person's point of view; de‐escalates tense situations; maintains working relationships; and promptly moves on from negative emotions
3. Professionalism: Exhibiting responsibility in their work, managing work effectively, maintaining a sustainable personal life and being prepared to meet job requirements, while adhering to professional and moral standards	
Ethics: Nurse is guided by moral principles in working and in interactions with others.	
Beginning	Nurse might not be fully aware of the professional code of ethics so may not fully execute it in practice; in witnessing unethical behaviour, may not respond at the moment and does not always take steps to address it
Developing	Nurse is aware of professional code of ethics and is able to apply it in practice; in witnessing unethical behaviour, responds in indirect ways such as seeking advice from a more experienced nurse
Accomplished	Nurse takes the initiative to resolve an ethical issue; may officially report the issue to superiors or the ethics committee if it cannot be resolved
Exemplary	Nurse consistently models ethical decision‐making based on the best interests of the patient and professional code of ethics; and is willing to take professional risks by directly addressing the issue with those involved
Dependability: Nurse shows reliability, takes responsibility for their actions and can be trusted to complete job tasks	
Beginning	Nurse is usually reliable in work, but may avoid aspects of care, be unsure of what to do or occasionally take shortcuts or blame others for not following instructions when performing care; and may occasionally be late for a shift.
Developing	Nurse usually completes assigned care but may need a reminder to complete care; will frequently ask the next shift to finish care if time runs out; is rarely late for a shift.
Accomplished	Nurse monitors work and completes all assigned care without a reminder; if necessary, will occasionally ask the next shift to finish care; is consistently on time.
Exemplary	Nurse autonomously completes all assigned care and explores any errors to prevent their recurrence; is accountable for own work, realistic about what she/he can commit to and anticipates where care will be needed; initiates new orders and proactively communicates with the next shift to promote optimal care. (continued)
Professional behaviour: Nurse's behaviour is appropriate for workplace situations; nurse complies with expectations for the professional role	
Beginning	Nurse may be unaware of workplace policies and may not provide nursing care based on institutional standards; nurse may follow others' examples in acting unprofessionally; nurse may be unreceptive to information about expectations for the professional role.
Developing	Nurse is aware of policies but may not consistently follow them; knows where to find policy statements but may not know how to interpret them; is becoming socialised to workplace norms for expected behaviour.
Accomplished	Nurse usually knows and follows policies and is receptive to new information about professional role expectations; may intervene to guide others in adhering to workplace expectations.
Exemplary	Nurse knows and consistently follows policies; seeks out policies to support decisions in unusual situations; contributes to a positive workplace culture by providing corrective/constructive feedback to others as needed.
Preparedness: Nurse anticipates job requirements and shows readiness to meet them	
Beginning	Nurse does not bring the necessary equipment/supplies to the job; and seems unaware of job expectations and requirements
Developing	Nurse brings enough of the basic equipment/supplies to the job to function adequately; and is becoming aware of job expectations and requirements
Accomplished	Nurse brings all the equipment/supplies to the job to function fully; plans ahead to exceed basic job expectations
Exemplary	Nurse brings additional tools to the job that may be needed based on assignment information; before initiating care, allows extra time to prepare self with information and supplies
Work management: Nurse uses strategies to manage own workday and patient care	
Beginning	Nurse has difficulty prioritising nursing care; creates an initial schedule in a linear fashion and may not be flexible or adapt to changes
Developing	Nurse is beginning to be able to prioritise; nurse can group tasks and organise care systematically and complete most care within the shift
Accomplished	Nurse is able to prioritise and plan a workflow to deliver all patient care; is able to consistently exceed bare minimum expectations without prompting; is able to rearrange schedule to accommodate patients' needs and get work done in a timely manner
Exemplary	Nurse is able to analyse several patient scenarios at one time; sets priorities and plans work to meet all patients' needs; and anticipates the need to rearrange the day based on emerging situations
Stress management: Nurse identifies and manages work‐related stress to maintain effectiveness in role	
Beginning	Nurse may be overwhelmed by stressful situations and may not admit to stress; lack of stress management may put self and patients at risk
Developing	Nurse is sometimes able to identify own stress but refrains from sharing it with others; attempts to find ways to manage stress without help
Accomplished	Nurse responds to stress by sharing it with others; develops stress management strategies; and applies them as needed
Exemplary	Nurse is able to avoid being overwhelmed by stressors; has developed effective coping mechanisms to help self and coworkers manage stress in order to maintain effectiveness in role
4. Teamwork: Showing full engagement in their work, assisting coworkers and making full use of resources while effectively leading the team.	
Resourcefulness: Nurse asks for help when necessary and is aware of and makes full use of available resources to overcome difficulties	
Beginning	Nurse may not ask for help or seek resources when help is needed; may frequently ask for help for something that could be done independently
Developing	Nurse may ask for appropriate help, but may wait until someone offers it; has basic knowledge of resources; and sometimes seeks information to expand knowledge
Accomplished	Nurse usually asks for specific help from more experienced personnel; knows about most resources and usually uses them in problem solving; shares resources to reach team solutions
Exemplary	Nurse recognises assignment overload and appropriately asks for help on high‐priority tasks; serves as a resource for others; and uses various resources effectively in problem solving
Support for coworkers:nurse shows willingness to assist staff in patient care duties and openly shares information	
Beginning	Nurse assists coworkers with patient care or information primarily when asked for specific help; may not volunteer to help coworkers; may not initiate care that would help the next shift.
Developing	Nurse occasionally offers patient care assistance or information to help coworkers without being asked.
Accomplished	Nurse willingly offers assistance to coworkers by providing patient care, sharing knowledge or helping them find information; looks for circumstances where help is needed.
Exemplary	Nurse anticipates when others may need assistance and manages own time to provide help; communicates willingness to help coworkers complete care as well as grow and develop; and mentors coworkers by sharing tips learned from own experience.
Professional involvement: Nurse actively contributes to job, healthcare team, organisation and profession	
Beginning	Nurse may join professional organisations but does not actively participate; does not actively contribute as part of a team
Developing	Nurse joins a unit‐based organisation and participates in unit activities but is passively engaged; does not take a leadership position; seeks some opportunities to contribute to the healthcare team
Accomplished	Nurse actively participates in unit‐based or local professional organisations and encourages others to join; offers insights on patient care as active member of the healthcare team
Exemplary	Nurse takes a leadership role in local or national organisations as a proactive voice for change; offers information to encourage adoption of current evidence‐based practices; and encourages teamwork, supports team members and values their input
Leadership: Nurse assumes responsibility for team problem solving and delegates care tasks to team members when appropriate	
Beginning	Nurse may delegate responsibilities (or care tasks) to lower‐level personnel but sometimes does not supervise them; follows rather than leads and tries to do everything alone
Developing	Nurse seeks guidance on how to use knowledge of team members' capabilities to delegate; begins to assign work to peers; may start to seek leadership opportunities
Accomplished	Nurse proactively takes on leadership roles; assigns work to meet goals according to the situation based on a sound rationale; provides enough information when delegating to ensure safety and efficiency
Exemplary	Nurse actively leads the team as a role model, mentor and supporter; considers the whole unit when making delegation decisions, appropriately assigns care tasks and follows up to ensure patient safety; shows professionalism and competence that maintain a positive environment for the unit as a whole
5. Caring role: Understanding and responding to others' needs and advocating for patients	
Responsiveness: Nurse identifies and responds appropriately to the emotional and physical needs of patients, families and their coworkers	
Beginning	Nurse may notice patients', families' and coworkers' needs but delays responding to them because of excessive focus on predetermined care plans
Developing	Nurse notices and responds to patients', families' and coworkers' needs but does not really connect with them emotionally
Accomplished	Nurse recognises and responds to changes in patients', families' and coworkers' needs and tries to solve the issues at hand; understands patients' and families' emotions and offers support
Exemplary	Nurse anticipates patients', families' and coworkers' needs and proactively addresses issues; makes meaningful connections with patients and families; and provides comfort and resources
Patience: Nurse can accept necessary delays and tedious/repetitive duties without undue frustration	
Beginning	Nurse easily becomes frustrated and upset when encountering delays and repetitive duties; might blame others for the situation
Developing	Nurse devotes a little time to respond to the situation but then takes over; remains externally calm during the situation but expresses negative feelings afterwards
Accomplished	Nurse devotes some time to respond to the situation and then intervenes; remains externally calm and professional throughout
Exemplary	Nurse devotes as much time as necessary to respond to the situation; remains understanding and encouraging without becoming annoyed; is willing to repeat explanations in simpler terms
Patient advocacy: When patients' safety, comfort or privacy is threatened, nurse takes action to ensure their interests are safeguarded	
Beginning	Nurse does not recognise situations when she/he should take action on behalf of patients; may not be sure of the appropriate action to take
Developing	Nurse sometimes recognises when a situation requires action; knows what to do; may not act at times because of lack of courage or because the patient is not the first priority
Accomplished	Nurse recognises when a problem or potential issue requires action; puts the patients' needs first and advocates on their behalf
Exemplary	Nurse critically thinks about patient issues; proactively and appropriately advocates for patients regardless of relationships with coworkers

### 3.1 Personal growth

*Personal growth* was characterised by an awareness of one's own interests and limitations in abilities, a commitment to learning and professional growth and demonstrating self‐confidence in abilities through independence. The subcompetencies of personal growth include self‐awareness, showing interest, professional development and self‐confidence (refer to Table 1).

The beginning level of personal growth was often characterised by nurses' not perceiving their abilities and limitations realistically. They might not show interest in improving their skills, potentially requiring prompts to engage in professional development opportunities. Additionally, they might need frequent and extensive direction. One interviewee commented that beginning‐level nurses do not yet prioritise personal growth, as they are ‘just trying to understand their job…as a nurse’ [C2]. The developing level of personal growth was described as nurses' beginning to articulate difficulties, not hesitating to ask questions, seeking out continuing education in their specialty to develop their practice and prevent errors and starting to establish a professional identity. Two participants stated that nurses at the developing level begin ‘to seek out training for things that are difficult for them’ [P9] and seek ‘professional development such as certifications or going to conferences’ [C3]. At the accomplished level of personal growth, nurses might identify professional resources and seek to utilise them, sharing what they learn and solutions to problems to improve the quality of care in their workplace. One participant commented that ‘[an accomplished nurse would] find resources to help [them learn] better…[and] outside education to help them to advance…in the profession’ [C2]. The exemplary level was described as nurses' being self‐directed and taking the initiative in requesting necessary work adjustments and professional development to enhance the unit. As one participant stated, ‘they [obtain] specific certifications pertinent to our floor; they may take additional roles’ [P1].

### 3.2 Effective interaction

*Effective interaction* entails exchanging information clearly and accurately, acting on constructive feedback and resolving conflicts. The subcompetencies within effective interaction include communication, feedback and conflict resolution (refer to Table 1). CNEs asserted that a nurse excels by engaging in clear and effective communication, actively seeking feedback from patients and staff to enhance performance and adeptly managing challenging situations with diplomatic conflict resolution.

CNEs described four levels of effective interaction. At the beginning level, nurses sometimes struggled with clear communication, effectively responding to feedback and managing conflict and emotions during challenging interactions. For example, one participant stated, ‘they might just not know that it's something they should report’ [P4]. The developing level was described as nurses effectively communicating patient conditions to the care team, trying to constructively respond to feedback for improvement and having developed strategies for managing emotions and quickly moving past conflicts when they are not effectively resolved. Another participant said, ‘they basically know a lot of ways to…talk to team members and a patient’ [P5]. The accomplished level of effective interaction was described as nurses' confidently communicating patient conditions with comprehensive details and suggestions, actively seeking and valuing feedback for practice improvement and directly and respectfully addressing conflicts, focusing on teamwork and maintaining professional relationships. One example of going beyond accurate reporting to offer solutions was described as ‘giving…a detailed suggestion based on…the scenario’ [P6]. Exemplary communication by nurses was described as communicating professionally and effectively to foster a positive culture for attainment of mutual goals, actively seeking and applying feedback for practice improvement without defensiveness and effectively resolving conflicts through empathy, de‐escalation, relationship preservation and quick recovery from negativity. An example was provided by one participant: ‘a nurse is able to break down medical terminology to…language that the patient can understand’ [P6].

### 3.3 Professionalism

*Professionalism* refers to nurses' adhering to professional and moral standards, exhibiting responsibility in their work, being prepared to meet job requirements, managing work effectively and maintaining a sustainable personal life. The subcompetencies within professionalism are ethics, dependability, professional behaviour, preparedness, work management and stress management (see Table 1). In this context, CNEs stated that a professional nurse is guided by ethical standards; demonstrates reliability, responsibility and trustworthiness; adheres to professional expectations; proactively meets job requirements; effectively organises their work and patient care; and manages stress to remain effective in their role.

Participants described four levels of nurses' professionalism. The beginning level of professionalism was described as follows: nurses are potentially unaware of professional ethics and workplace policies, may execute care inconsistently and might not always adhere to standards or manage stress effectively, thereby risking both personal and patient safety. One participant stated, ‘they just try to stay in the same order all the time’ [P3]. At the developing level, a nurse is aware of the professional code of ethics and familiar with policies and is learning job expectations but is not always consistent in following or completing them; at the same time, they are improving in prioritising care and beginning to manage stress. Another participant said about the developing level, ‘[nurses are] starting to develop the appropriate time management skills’ [P2] and ‘customize the workflow…that would suit them the best’ [C4]. The accomplished level of professionalism was described as a nurse proactively addressing ethical issues, adhering to and enforcing policies, fully preparing with necessary supplies to surpass expectations, skilfully prioritising patient care with adaptability, reliably completing and monitoring care and effectively managing stress through such strategies. One participant offered this example: ‘You'd have your customized system in place, you'd know what works best for you…, you will feel more confident, it will take less time to prioritize and organize’ [C4]. The exemplary nurse's professionalism was described as follows: a nurse exemplifies ethical decision‐making aligned with patient best interests, adheres to and seeks policies to make informed decisions, prepares thoroughly with necessary tools and information, autonomously completes care while addressing errors and anticipates needs, efficiently analyses and prioritises multiple patient needs and successfully manages stress with developed coping mechanisms to maintain role effectiveness. According to one participant, ‘your system is completely in place, you have full confidence that it is the best way…for you’ [C4].

### 3.4 Teamwork

*Teamwork* refers to nurses' showing full engagement in their work, assisting coworkers and making full use of resources while effectively leading the team. Four subcompetencies within the teamwork competency were identified by participants: support for co‐workers, resourcefulness, professional involvement and leadership (see Table 1). In the teamwork context, a nurse proactively seeks assistance, utilises resources effectively, collaborates and shares information willingly, contributes actively to their job and the broader healthcare context and assumes responsibility for team problem solving and delegating tasks appropriately.

Interviewees stated that at the beginning level of teamwork, a nurse may hesitate to seek help or use resources when working independently, assists coworkers when asked, joins but rarely participates in professional organisations and often follows rather than leads. Two participants stated that ‘beginning nurses don't like to do that [ask for help] because they think that will make them look…incompetent’ [P3] and that ‘[beginning nurses ask] questions on things I know they know how to do’ [P7]. At the developing level, a nurse sometimes asks for help or uses resources after they are offered, offers assistance to coworkers unprompted, joins and passively participates in unit activities without seeking leadership roles and is beginning to learn how to delegate effectively and explore leadership opportunities. Participants described this level as ‘making people aware that you're having a hard time so they can…help you’ [P2] and ‘starting to ask [for] help on things that may be a little more important’ [P7]. At the accomplished level, a nurse seeks experienced help, leverages and shares resources, assists with and anticipates coworker needs, engages in professional organisations and assumes leadership, strategically delegating tasks for safety and efficiency. One participant illustrated the accomplished level as ‘having more knowledge on the resources and where to find things [and] who to contact for certain things’ [C4]. At the exemplary level, a nurse adeptly manages assignment overload by seeking help, acts as a proactive resource and mentor, takes leadership roles and advocates for evidence‐based change and leads by example with strategic delegation and support, fostering a positive and professional unit environment. As an example, exemplary nurses ‘know who their resources are. They know how to…get assistance as needed to take care of the patients’ [P9].

### 3.5 Caring role

*Caring role* is defined as understanding and responding to others' needs and advocating for patients. The three subcompetencies within the caring role competency were responsiveness, patience and patient advocacy (see Table 1). Specifically, a nurse adeptly identifies and meets the emotional and physical needs of patients, families and coworkers; accepts delays and repetitive tasks without frustration; and proactively safeguards patients' safety, comfort and privacy when threatened.

At the beginning level of caring role, a nurse might notice but delay responding to the needs of patients, families and coworkers due to a rigid focus on care plans; becomes easily frustrated with delays and repetitive tasks; and may not recognise or know how to act in situations requiring advocacy for patients. One participant stated, ‘you are noticing those patients' needs but maybe you're not always acting on them’ [C2]. At the developing level, a nurse notices and addresses the needs of patients, families and coworkers without forming emotional connections; responds with surface‐level calmness while harbouring negative feelings; and despite recognising necessary actions, may hesitate due to lack of courage or poor prioritisation. One participant said of this level, ‘[nurses] can't respond to them because they …are someone else's patients’ [C2]. The accomplished level of caring role was described as a nurse's recognising and addressing the evolving needs of patients, families and coworkers; offering emotional support; responding with calm professionalism; and prioritising patients' needs, advocating for them when necessary. Another participant illustrated the accomplished level as follows: ‘she answers the call [for] aid, solves the issue [to the] best [of] her ability…and notifies me and clarifies what she did, asking if this was done correctly’ [C4]. The exemplary level was described as a nurse proactively anticipating and meeting the needs of patients, families and coworkers; forging meaningful connections and providing comfort; spending ample time addressing situations with patience and encouragement; and critically advocating for patients effectively, regardless of coworker relationships. As an example of the exemplary nurse's responsiveness, a participant said that ‘if an IV pump is beeping, even if it is not yours, you… go look for [it]… You understand what the priority is, and you're paying attention to your surrounding area’ [P6].

## 4 DISCUSSION

This study identified critical soft skill competencies that CNEs consider important to evaluate in nurses along with four levels of nursing behavioural indicators that reflect their current status.

With respect to critical soft skills to be evaluated in nurses, we attempted to expand the findings for critical soft skill competencies beyond the self‐perceived competencies revealed in a previous literature review (Song & McCreary, 2020) by exploring CNEs' perspectives. Additionally, the previous literature review was necessarily based on past phenomena in the nursing field, while our current study's findings reflect more recent trends and practical needs at the bedside. In completing this study, we have attempted to narrow the gap between clinical education and practice requirements while also strengthening the usefulness of the rubric to be developed.

Furthermore, based on the findings, objective behaviour indicators for four skill levels of these competencies were created by drawing upon the clinical nursing education expertise of nurses with different practice backgrounds, thus providing a framework for the development of a soft skill assessment rubric that is both summative and formative in nature. We believe that the comprehensive behavioural indicators, levels and other findings generated in this study can guide constructive feedback and enhance the future rubric's applicability for nurses at all levels, helping them identify concrete goals for improving their practice. We acknowledge that because most competency assessments are conducted during the first years of practice, NGNs would be the principal beneficiaries of this study's findings.

CNEs identified four subcompetencies that comprise the *personal growth* competency: self‐awareness, showing interest, professional development and self‐confidence.

The value of these findings aligns with the codes of ethics of the American Nurses Association [ANA] (2015) and the International Council of Nurses [ICN] (2021). These codes emphasise the importance of maintaining competence and fitness to practice through continuous personal and professional growth to ensure that the quality of care is not compromised. Additionally, the four levels' descriptions of developmental progression in personal growth demonstrate how a nurse's evolving abilities and skills can contribute to increased self‐confidence and improved quality of care. Despite the importance of personal growth, organisations' failure to recognise nurses' personal needs and to provide accessible resources were shown as key barriers (Hakvoort et al., 2022). To motivate nurses to engage in professional growth activities, employers could tailor these opportunities to match the nurses' individual learning needs and interests. This may enhance nurses' retention at hospitals (Vázquez‐Calatayud et al., 2021).

As to the *effective interaction* competency, CNEs identified three subcompetencies: communication, feedback and conflict resolution. Collectively, these qualities embody diplomacy, which epitomises effective interaction. Moreover, the levels' descriptions of developmental progression in effective interaction reflect the CNEs' emphasis on the art of dealing with patients, families and colleagues sensitively and effectively in particular clinical contexts. These subcompetencies are consistent with a previous study involving critical care nurses, who perceived diplomacy as an essential element of their educational requirements (Currey et al., 2022). Previous studies reported that nurses in general expressed confidence in the effectiveness of their usual communication (Leal‐Costa et al., 2020; Walton et al., 2018). However, consistent with the CNEs' observations, one area of concern is managing conflict appropriately and professionally, especially with team members from other disciplines (Somani et al., 2021). These findings suggest that nurses should receive enhanced training opportunities facilitated by technologies such as simulation or virtual reality to practice diplomatic communication skills in role playing exercises and conflict resolution conversations (Choi et al., 2021; Hoek et al., 2023).

With respect to the *professionalism* competency, CNEs identified six subcompetencies: ethics, dependability, professional behaviour, preparedness, work management and stress management. These reflect CNEs' attention to the value of trust, integrity, expertise and judgement, all of which align with the codes of ethics of ANA (2015) and ICN (2021). The codes stipulate that nurses must uphold their professional integrity and practice self‐care to preserve their character, thus empowering them to make decisions as autonomous professionals in order to ensure the highest quality of care. As for the levels' descriptions of developmental progression in professionalism, they represent incremental mastery of these subcompetencies as nurses become aware of professional ethics and policies, learn to follow them in practice and simultaneously pursue autonomy. It is encouraging that, due to their continuous pursuit of an autonomous professional identity, nurses have earned a high degree of respect from the public, surpassing many other professions in this regard (Godsey et al., 2020). Nevertheless, the nursing workforce shortage persists and one factor contributing to this shortage is closely linked to nurses' perceptions of inadequate professional autonomy (van der Cingel & Brouwer, 2021). Research indicates that more fully addressing this core value in practice could prevent nurses from leaving the profession due to feelings of unfulfillment (Blay & Smith, 2020).

Under the *teamwork* competency, CNEs identified four subcompetencies: support for coworkers, resourcefulness, professional involvement and leadership. CNEs highlighted the value of proactive collaboration because the nature of nursing practice is inherently team based, and its success hinges on teamwork. This perspective aligns with elements identified in previous studies, which emphasise the importance of nurturing, accepting the responsibility to lead and demonstrating sensitivity to emerging needs (Whitehair et al., 2018). As the levels describe, developmental progression occurs as nurses evolve—that is, as they learn to manage workloads through strategic delegation and seeking help, serve as proactive mentors, advocate for evidence‐based change and lead by example, thereby fostering a positive, professional unit environment.

Consistent with these descriptions, earlier research indicated that NGNs, who are typically at a developmental stage in their careers, expressed a need to improve their overall teamwork and leadership skills (Burger et al., 2010; Dyess & Sherman, 2011; Walton et al., 2018). In contrast, nurses with more experience generally regarded their teamwork and leadership skills as acceptable (Castner et al., 2012). To better prepare NGNs for these challenges, orientation and clinical education could be enriched to include more hands‐on experience in collaborating with other nurses and with personnel outside their discipline.

Finally, the *caring role* competency consists of responsiveness, patience and patient advocacy as subcompetencies. Given that caring is both the identity and the soul of the nursing profession (Bratajaya, 2021), the CNEs emphasised nurses' internalisation of the value of caring, which is manifested as attentiveness to the needs of patients, family members and coworkers. This finding is again consistent with the ANA (2015) and ICN (2021) codes of ethics. The levels of caring role behaviours are gradated from the beginning nurse who delays responding to needs due to a focus on their own tasks, who becomes frustrated with their duties and who does not take action on behalf of patients, to the exemplary nurse who anticipates needs, devotes sufficient time to responding to situations and advocates for patients by prioritising them. These findings align with a previous study in which nurses expressed a need to better manage their emotional reactions in the workplace and cultivate their patient advocacy skills (Burger et al., 2010; Walton et al., 2018). To facilitate growth of these intangible attributes, nurses could be exposed to means of managing demanding situations through role playing, case conferencing or simulation (Walton et al., 2018). At the same time, nursing leadership could help nurses understand that serving as an advocate for patients' best interests is a cultural expectation (Hunter & Cook, 2018).

This study's limitations should be noted. As it was conducted in a single hospital, the findings may have limited transferability. This limitation was lessened by interviewing CNEs from multiple units to gather a variety of perspectives. Additionally, limitations in interpreting the interviewees' spoken statements may have arisen. This issue was mitigated first through immediate member checking of interviewee responses during data collection and then through a coauthor's participation in and review of the data analysis process to confirm the first author's judgements.

### 4.1 Implications for nursing and health policy

Despite the importance of soft skill competencies in nursing, their intangible nature has led to problems in evaluating their attainment by nurses. Application of our findings in healthcare facilities and clinical nursing education programmes could guide evaluation of these neglected but critical skills in practicing nurses. Moreover, our findings could support development and implementation of improved strategies for facilitating NGNs' transition to workplace processes, nurse development and service monitoring as well as nurse retention. Furthermore, key stakeholders in clinical nursing education could use our findings to define soft skill competency standards for practice and could initiate dialogues for formal integration of these standards into the healthcare system.

## 5 CONCLUSION

This study enhances understanding of CNEs' perceptions of critical soft skill competencies given the job requirements faced by nurses. As to those competencies, perception gaps between these experienced personnel and nurses argue for the need for clear performance standards as well as an objective assessment tool to guide nurses' improvement of their competencies. Application of such a tool would allow academic and clinical educators to better guide NGNs and other nurses in improving their soft skills and would contribute to comprehensive preparation and improved retention of nurses. In the research realm, future studies should explore associations between soft skill development and individual, team and organisational factors and should assess the impact of soft skill development on nurse retention.

## AUTHOR CONTRIBUTIONS

Study design: YS, CML, CV, MJK, CGP and LLM. Data collection: YS. Data analysis: YS and LLM. Study supervision: CML and LLM. Manuscript writing: YS, CML, CV, MJK and LLM. Critical revisions for important intellectual content: YS, CML and LLM.

## FUNDING INFORMATION

This work was supported by the University of Illinois at Chicago Provost's Graduate Research Award; Sigma‐Alpha Lambda Research Award; Seth and Denise Rosen Memorial Research Award; and the University of Illinois at Chicago College of Nursing PhD Student Research Award. This study was approved by the Office of the Protection of Research Subjects, University of Illinois at Chicago and the Nursing Research Council, University of Chicago (IRB19‐0395).

## CONFLICT OF INTEREST STATEMENT

No conflict of interest has been declared by the authors.

## Data Availability

The data that support the findings of this study are available from the corresponding author upon reasonable request.
